# Support vector machine-driven Parkinson’s disease identification: a 7-Tesla multidimensional structural MRI approach

**DOI:** 10.1038/s41531-026-01370-3

**Published:** 2026-04-29

**Authors:** Yongqin Xiong, Zhixuan Li, Mingliang Yang, Haoxuan Lu, Caohui Duan, Song Wang, Xiaoyu Wang, Jiayu Huang, Yan Li, Xi Yin, Yuchen Guo, Zhongbao Gao, Xin Lou

**Affiliations:** 1 Department of Radiology, Chinese PLA General Hospital, Beijing, China; 2 College of Medical Technology, Beijing Institute of Technology, Beijing, China; 3 Department of Neurology, the Second Medical Center & National Clinical Research Center for Geriatric Disease, Chinese PLA General Hospital, Beijing, China; 4 Beijing National Research Center for Information Science and Technology, Tsinghua University, Beijing, China

**Keywords:** Neurology, Biomarkers, Diagnostic markers, Diseases of the nervous system

## Abstract

Parkinson’s disease (PD) is a progressive neurodegenerative disorder diagnosed clinically by cardinal motor symptoms, with structural brain changes associated with its diverse motor and non-motor manifestations. This study integrated multidimensional 7-Tesla structural Magnetic Resonance Imaging (MRI) features (gray matter volume, cortical thickness, etc.) using Support Vector Machine (SVM) to distinguish 98 PD patients from 74 healthy controls. The SVM model achieved 0.80 accuracy (sensitivity: 100%, F1-score: 0.85) and identified key biomarkers. Partial Least Squares Regression (PLSR) revealed these features correlated significantly with motor symptoms (Movement Disorder Society-Unified Parkinson’s Disease Rating Scale [MDS-UPDRS]-III, tremor, rigidity, bradykinesia, postural instability; *P* < 0.05) and non-motor symptoms (cognition, anxiety, depression, MDS-UPDRS-I; *P* < 0.05). The findings highlight the potential of 7-Tesla MRI and machine learning as diagnostic tools for PD, while also providing insights into its pathophysiology. This approach may aid in detection and understanding of PD’s motor and non-motor manifestations.

## Introduction

Parkinson’s disease (PD) is a progressive neurodegenerative disorder, manifesting through motor symptoms such as bradykinesia, rigidity, and tremor, along with non- motor manifestations like cognitive decline and mood disturbances^1^. With more than 10 million people affected worldwide, PD poses a significant socioeconomic burden^2,3^. Pathologically, the disease is marked by the degeneration of dopaminergic neurons in the substantia nigra^4,5^. Beyond well-characterized dopaminergic dysfunction within the basal ganglia, extensive evidence confirms that PD is a multisystem neurodegenerative disorder with early involvement of multiple non-dopaminergic neurotransmitter systems (including cholinergic, serotonergic, and noradrenergic pathways) and widespread disruption of interconnected neural networks throughout the brain^6–10^. This multisystemic pathology underlies the broad spectrum of motor and non-motor symptoms in PD and drives structural and functional alterations in cortical regions far beyond the classical cortico-basal ganglia circuitry, highlighting the need to investigate global cortical morphological changes to fully capture PD-related neuropathology.

Ultra-high-field 7-Tesla (7 T) magnetic resonance imaging (MRI) has emerged as a revolutionary tool for investigating multidimensional morphological changes in the brain, with its exceptional spatial resolution enabling the detection of subtle microstructural changes^11^. Reductions in gray matter volume (GMV) and abnormalities in cortical thickness (CT) have been identified in PD, both structural features may be modulated by disease severity and associated with motor and non-motor symptoms^12–15^. Compared with GMV and CT, curvature and folding index not only characterize cortical surface morphology but also exhibit higher accuracy and sensitivity in aging populations, which may offer a novel perspective for investigating the neuropathological mechanisms underlying PD^13,16,17^. However, most previous studies based on the aforementioned structural MRI features have focused on group-level comparisons of structural differences between PD patients and healthy controls, which limits their utility for individualized clinical diagnosis.

Machine learning techniques have shown substantial advantages in assisting individualized diagnosis across various neurological and psychiatric disorders, including Alzheimer’s disease, schizophrenia, depression, and PD^18–23^. Compared with traditional group-level analysis, machine learning approaches could integrate high-dimensional neuroimaging data and take interregional correlations into account, thereby yielding higher sensitivity to subtle structural alterations and spatial distribution differences. For example, support vector machine (SVM) classifiers based on cortical surface area or thickness measurements have demonstrated favorable performance in differentiating schizophrenia patients from healthy controls^21^. Another SVM integrating cortical thickness and sulcal depth metrics achieved reliable diagnostic performance in identifying mild cognitive impairment due to Alzheimer’s disease^19^. In addition, using GMV, CT, local gyrification index, and local fractal dimension derived from 3 T structural MRI across several PD-related brain regions, Ya et al. constructed a logistic regression classifier that achieved an area under the curve (AUC) of 0.78 in distinguishing PD patients from healthy controls^20^. However, research investigating the diagnostic utility of 7 T MRI-derived multidimensional cortical metrics for PD remains limited.

Therefore, in this study, by using structural 7T MRI features, we extract multidimensional morphological features, including GMV, cortical thickness, curvature, surface area, and folding index of cerebral cortex, and constructed SVM model to investigate their diagnostic utility in patients with PD. We hypothesize that an SVM model based on multidimensional cortical structural features will yield favorable classification performance for PD. Moreover, we correlate structural alterations with clinical measures (e.g., Movement Disorder Society-Unified Parkinson’s Disease Rating Scale (MDS-UPDRS) Part III score) to clarify their relationship with the severity of motor and non-motor symptoms. By integrating ultra-high-resolution imaging with a multidimensional machine learning framework, this research promotes the development of robust, pathophysiological biomarkers for PD.

## Results

### Demographic and clinical evaluation

The demographic and clinical characteristics of the study participants, comprising 98 PD patients (mean age: 63.76 ± 9.21 years; 48 males) and 74 HCs (mean age: 64.85 ± 6.09 years; 27 males), are detailed in Table 1. The PD group had an average disease duration of 4.21 ± 2.92 years, with a mean levodopa equivalent daily dose (LEDD) of 454.03 ± 209.12 mg. 82.7% of patients were classified as Hoehn–Yahr stage ≤2.0. Statistical analyses revealed no significant differences between the PD and HC groups in terms of age (T = 0.89, *P* = 0.38) and gender distribution (χ^2^ = 2.68, *P* = 0.10), indicating that these variables were well-matched between the two groups. PD patients exhibited significantly lower MMSE scores and significantly higher HAMA and HAMD scores compared to the HC group (all *P* < 0.001). Additionally, the PD group showed notable motor impairments, as evidenced by the MDS-UPDRS III score (25.17 ± 10.89), with specific issues in bradykinesia, rigidity, tremor and PIGD.

**Table 1:** Demographic and clinical data of participants

Variables	HC (*N* = 74)	PD (*N* = 98)	Statistics	*P*-value
Gender (M/F)	27/47	48/50	χ^2^ = 2.68	0.10
Age (years)	64.85 ± 6.09	63.76 ± 9.21	T = 0.89	0.38
Disease duration (years)	/	4.21 ± 2.92	/	/
LEDD (mg/day)	/	454.03 ± 209.12	/	/
**Motor manifestations**				
H-Y stage	/	1.71 ± 0.68	/	/
MDS-UPDRS III	/	25.17 ± 10.89	/	/
Tremor	/	4.46 ± 4.04	/	/
Rigidity	/	6.29 ± 3.21	/	/
Bradykinesia	/	9.69 ± 5.66	/	/
PIGD	/	2.57 ± 2.28	/	/
**Non-motor manifestations**				
MDS-UPDRS I	/	8.09 ± 5.23	/	/
MMSE^a^	28.68 ± 1.54	26.53 ± 3.93	U = 4346.50	4.76e-05
HAMA^b^	1.65 ± 2.15	6.01 ± 6.74	U = 1319.00	3.16e-08
HAMD^c^	1.34 ± 1.77	5.25 ± 5.61	U = 1505.00	2.06e-06

For continuous variables, data are presented as mean ± standard deviation, and comparisons are made using a two-sample t-test for normally distributed data and Mann-Whitney U test for non-normally distributed data. For qualitative variables, data are presented as frequency, and comparisons are made using the chi-squared test.

*HC* healthy control, *PD* Parkinson’s disease, *M* male, *F* female, *LEDD* levodopa equivalent daily dose, *H-Y* Hoehn–Yahr, *MDS-UPDRS* Movement Disorder Society-Unified Parkinson’s Disease Rating Scale, *PIGD* postural instability/gait difficulty, *MMSE* Mini-mental State Examination, *HAMA* Hamilton Anxiety Scale, *HAMD* Hamilton Depression Rating Scale.

^a^12 data were missing in PD group, results of 86 ePD and 74HC.

^b^24 data were missing in PD group, results of 74 ePD and 74HC.

^c^25 data were missing in PD group, results of 73 ePD and 74HC.

### Support vector machine

**Fig. 1:**
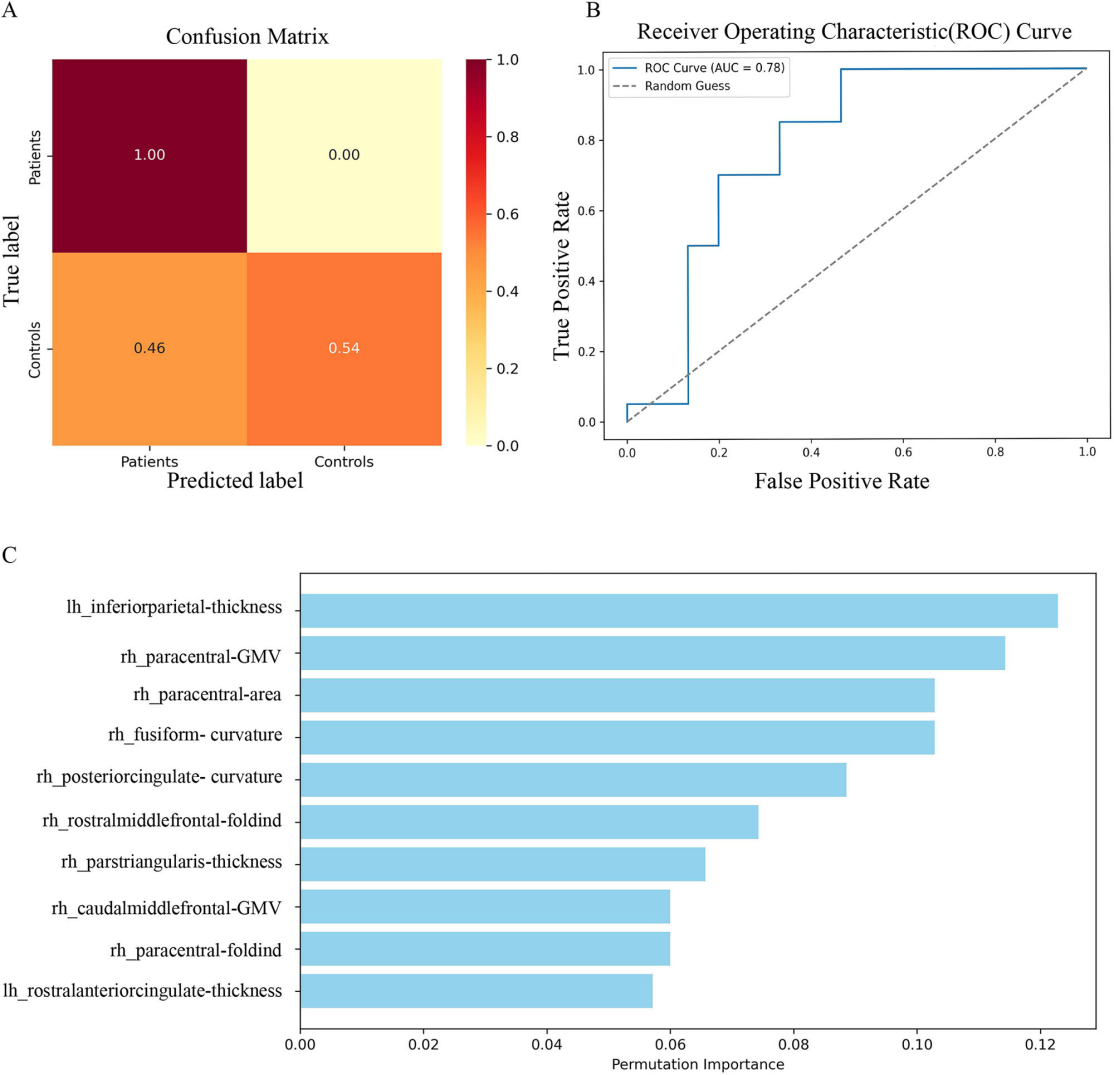
SVM classification based on multidimensional cortical indicators. **A** Convolution maps of the SVM. **B** Receiver operating characteristic (ROC) curve. **C** Top 10 features based on the permutation importance.

Mixed-effects linear regression models identified 13 GMV, 5 surface area, 7 CT, 3 curvature, and 7 folding index features for subsequent model development (see Supplementary Table S2 for details). Using these identified features, we built separate SVM models for each morphological metric (GMV, surface area, CT, curvature, and folding index), as well as a combined model integrating all features. When evaluated individually, these single-metric SVM models achieved classification accuracies of 0.66, 0.66, 0.60, 0.54, and 0.63 for GMV, surface area, CT, curvature, and folding index, respectively. In contrast, the SVM model incorporating the combined multidimensional structural MRI metrics, optimized with a radial basis function (RBF) kernel (hyperparameters: C = 1, γ = 0.01), demonstrated superior performance. The integrated SVM model achieved an overall accuracy of 0.80, defined as the ratio of correctly predicted instances to the total number of predictions. Additionally, in the test set, this model achieved a sensitivity of 100.0%, precision of 74.0%, and F1-score of 0.85 for PD patients, as well as a sensitivity of 54.0%, precision of 100.0%, and F1-score of 0.70 for HC; the area under the receiver operating characteristic (ROC) curve (AUC) value of the model was 0.78 (Fig. 1A, B). The macro-average and weighted-average scores were 0.87 and 0.85, respectively, indicating robust performance across multiple evaluation metrics. Furthermore, the fold-wise performance obtained during training is presented in Supplementary Table S3. In the subsequent permutation importance analysis, the cortical thickness of the left inferior parietal lobule (feature importance = 0.123), GMV of the right paracentral gyrus (0.114), and surface area of the right paracentral gyrus (0.103) emerged as the top three influential predictors (Fig. 1C, Table 2).

**Table 2:** The top 10 features of SVM based on permutation importance

Ranking	Feature	Permutation importance
1	lh_inferiorparietal-thickness	0.123
2	rh_paracentral-GMV	0.114
3	rh_paracentral-area	0.103
4	rh_fusiform- curvature	0.103
5	rh_posteriorcingulate- curvature	0.089
6	rh_rostralmiddlefrontal-foldind	0.074
7	rh_parstriangularis-thickness	0.066
8	rh_caudalmiddlefrontal-GMV	0.060
9	rh_paracentral-foldind	0.060
10	lh_rostralanteriorcingulate-thickness	0.057

*GMV* gray matter volume, *foldind* folding index, *rh* right hemisphere, *lh* left hemisphere.

### Correlation analyses of multidimensional cortical indicators and motor symptoms

**Fig. 2:**
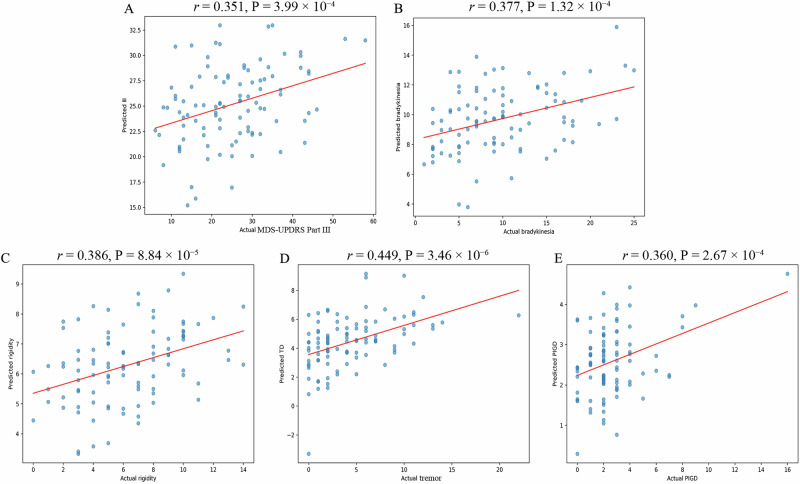
Scatter plots between important features derived from SVM model and motor symptoms in patients. **A** MDS-UPDRS Part III. **B** Bradykinesia. **C** Rigidity. **D** Tremor. **E** postural instability/gait difficulty (PIGD).

The PLSR analyses revealed statistically significant associations between SVM-derived features with permutation importance >0.01 and motor symptoms. Specifically, single-component models achieved strong predictive power for MDS-UPDRS Part III score (F = 13.47, *P* = 3.99 × 10^−4^), bradykinesia (F = 15.86, *P* = 1.33 × 10^−4^), rigidity (F = 16.76, *P* = 8.84 × 10^−5^) and postural instability/gait difficulty (F = 14.33, *P* = 2.27 × 10^−4^). For tremor severity, a two-component model was optimal (F = 12.02, *P* = 1.19 × 10^−5^). Pearson correlation analyses confirmed significant linear associations between predicted and observed scores: MDS-UPDRS Part III (r = 0.351, *P* = 3.99 × 10^−4^), bradykinesia (r = 0.377, *P* = 1.32 × 10^−4^), rigidity (r = 0.386, *P* = 8.84 × 10^−5^), tremor (r = 0.449, *P* = 3.46 × 10^−6^), and PIGD (r = 0.360, *P* = 2.67 × 10^−4^) (Fig. 2A–E).

The VIP analysis, a critical step in PLSR for identifying dominant predictors, highlighted neuroanatomical features driving these associations, with a threshold above >1.5 reflecting significant contribution. For H-Y stage, the left cuneus surface area (VIP = 1.959), left lateral occipital folding index (VIP = 1.943), and right fusiform gyrus mean curvature (VIP = 1.931) emerged as the top predictors. For MDS-UPDRS Part III scores, the right banks of the superior temporal sulcus thickness (VIP = 2.200), right precentral gyrus volume (VIP = 1.634), and left inferior parietal thickness (VIP = 1.521) were prioritized, aligning with motor execution pathways. For bradykinesia, the left lateral occipital folding index (VIP = 2.209), right banks of the superior temporal sulcus thickness (VIP = 2.012), and left inferior parietal thickness (VIP = 1.602) were identified as key drivers. For rigidity, the left lateral occipital folding index (VIP = 1.594), right middle temporal volume (VIP = 1.592), and right lingual gyrus volume (VIP = 1.562) were most influential. For tremor, the right posterior cingulate mean curvature (VIP = 2.531), left inferior parietal thickness (VIP = 2.390), and right pars opercularis thickness (VIP = 2.232) dominated predictions. Finally, for PIGD, the right fusiform mean curvature (VIP = 1.854), right banks of the superior temporal sulcus thickness (VIP = 1.740), and left inferior temporal volume (VIP = 1.707) were prioritized. See Supplementary Table S4 for further details.

### Correlation analyses of multidimensional cortical indicators and non-motor manifestations

**Fig. 3:**
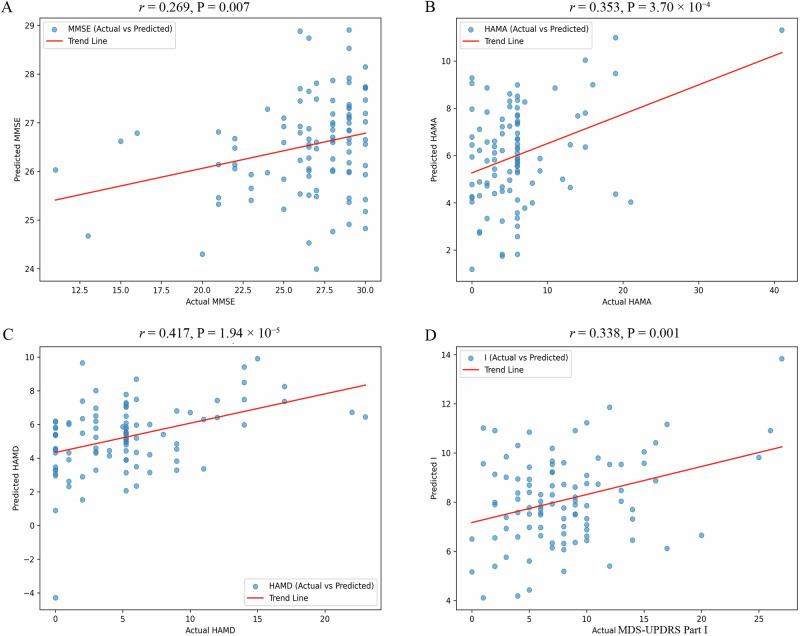
Scatter plots between important features derived from SVM model and non-motor manifestations in patients. **A** MMSE. **B** HAMA. **C** HAMD. **D** MDS-UPDRS Part I.

The PLSR models demonstrated robust predictive accuracy across multiple non-motor manifestations, with distinct dimensionality configurations for each clinical measure. A single-component model exhibited significant predictive power for the MMSE (F = 7.89, *P* = 0.007) and the HAMA (F = 13.63, *P* = 3.70 × 10^−4^), while a two-component model best captured variance in the HAMD (F = 10.00, *P* = 1.15 × 10^−4^). Similarly, a single-component model showed strong performance for the MDS-UPDRS Part I score (F = 12.36, *P* = 6.72 × 10^−4^). Pearson correlation analyses confirmed significant linear associations between predicted and observed scores: MMSE (r = 0.269, *P* = 0.007), HAMA (r = 0.353, *P* = 3.70 × 10^−4^), HAMD (r = 0.417, *P* = 1.94 × 10^−5^), and MDS-UPDRS Part I (r = 0.338, *P* = 0.001) (Fig. 3A–D).

Variable Importance in Projection (VIP) analyses identified domain-specific neuroanatomical predictors (VIP > 1.5). For MMSE, the right pars opercularis cortical thickness (VIP = 2.008), right medial orbitofrontal volume (VIP = 1.926), and left inferior parietal thickness (VIP = 1.713) emerged as dominant predictors. For HAMA, the right fusiform gyrus mean curvature (VIP = 2.322), left inferior temporal volume (VIP = 1.905), and left rostral anterior cingulate thickness (VIP = 1.538) were identified as key drivers. For HAMD, the left precuneus volume (VIP = 2.416), left rostral anterior cingulate thickness (VIP = 2.109), and right parahippocampal folding index (VIP = 1.857) were prioritized. Finally, for MDS-UPDRS Part I, the right fusiform curvature (VIP = 2.453), right medial orbitofrontal volume (VIP = 1.689), and left entorhinal thickness (VIP = 1.680) were most influential. See Supplementary Table S5 for further details.

## Discussion

This study leveraged ultra-high-field 7 T MRI and an SVM model to integrate multiple morphological metrics—GMV, CT, surface area, curvature, and folding index—for the classification of PD patients and HC. The multidimensional model achieved an overall accuracy of 0.80, significantly outperforming the single-parameter models, which yielded accuracies ranging from 0.54 to 0.66. Permutation importance analysis identified the cortical thickness of the left inferior parietal lobule, GMV of the right paracentral gyrus, and surface area of the right paracentral gyrus as the most influential predictors, aligning with established PD-related neuroanatomical changes^24–27^. Furthermore, PLSR analyses revealed significant associations between specific neuroanatomical features and both motor and non-motor functions in PD, providing novel insights into the structural correlates of PD manifestations. These results highlight the potential of advanced neuroimaging combined with machine learning to provide pathophysiological biomarkers for PD identification.

This study employed ultra-high-field 7 T MRI combined with an SVM model to integrate five distinct morphological metrics—GMV, CT, surface area, curvature, and folding index—for distinguishing between PD patients and HC. The multidimensional integrated model yielded an overall accuracy of 0.80, which outperformed the single-parameter SVM models established in the same study. These single-metric models were trained on individual morphological parameters using the identical 7 T MRI dataset and analytical pipeline, and only achieved moderate accuracy ranging from 0.54 to 0.66. This within-study enhancement in performance underscores the advantage of integrating multiple morphological features, as PD pathology involves complex, multifaceted structural alterations across cortical domains that cannot be adequately captured by isolated metrics^20,22,28^. For instance, while GMV provides insights into volumetric changes, with multiple studies reporting significant GMV reductions in PD patients’ prefrontal regions compared to controls^14^, cortical thickness and surface area offer complementary information on cortical integrity and complexity, with folding index and curvature reflecting distinct aspects of cortical gyrification and morphological geometry^15,16^.

Notably, previous studies employing 3 T MRI-derived multidimensional cortical metrics for the classification of neurodegenerative diseases, such as PD (using CT, GMV, local gyrification index, and local fractal dimension^20^) and mild cognitive impairment due to Alzheimer’s disease (using cortical thickness and sulcal depth^19^), have demonstrated reliable classification performance. However, these investigations were largely restricted to conventional 3 T MRI, which may lack the resolution necessary to capture subtle cortical alterations in PD. In contrast, our work is the first to integrate five distinct morphological metrics from ultra-high-field 7 T MRI for PD classification; 7 T MRI’s unprecedented spatial resolution and contrast-to-noise ratio enable the detection of subtle PD-related cortical structural changes that are undetectable by 3 T scanners^11,15^, which complements the multi-feature framework and underpins the diagnostic performance of our model for PD. Permutation importance analysis identified the cortical thickness of the left inferior parietal lobule (feature importance = 0.123), GMV of the right paracentral gyrus (0.114), and surface area of the right paracentral gyrus (0.103) as the most influential predictors in our SVM model. These findings align with established PD-related neuroanatomical changes, including cortical thinning in associative regions and sensorimotor regions^24–28^. The inferior parietal lobule, a key node in the visuospatial and sensorimotor networks, has been consistently implicated in PD-related motor and cognitive deficits^24,25^. Similarly, the paracentral gyrus—a key region for motor execution and coordination—exhibits pathological involvement in PD due to its connectivity with subcortical and cortical motor networks^26,27^.

The PLSR analyses in this study revealed robust associations between SVM-derived neuroanatomical features and PD motor symptoms, demonstrating the effectiveness of integrating machine learning with neuroimaging biomarkers for decoding complex neurodegenerative processes. The VIP analysis further identified region-specific morphological predictors for different motor domains, providing novel insights into the neural substrates of PD symptomatology. The severity of motor symptoms, quantified using the MDS-UPDRS Part III score, correlated with structural modifications in the cuneus area, lateral occipital folding index, fusiform gyrus curvature, banks of the superior temporal sulcus and inferiorparietal lobule thickness, as well as GMV alterations in the superior temporal gyrus, precuneus, rostral middle frontal gyrus, precentral gyrus, paracentral lobule, medial orbitofrontal cortex, caudal middle frontal gyrus, and middle temporal gyrus. These findings align with prior studies that demonstrated widespread structural changes in frontal, occipital, and temporal brain regions among PD patients^28–30^. The observed patterns of neurodegeneration correspond to the established caudo-rostral progression outlined in Braak staging, where advanced PD pathology (Braak stages V–VI) extends beyond nigrostriatal dopaminergic deficits to involve neocortical networks^5^.

The right banks of the superior temporal sulcus thickness, right precentral gyrus GMV, and left inferior parietal thickness emerged as primary predictors of bradykinesia severity. The banks of the superior temporal sulcus and inferior parietal lobule play critical roles in multisensory integration, sensorimotor feedback processing, and cross-modal associative functions^31,32^. Meanwhile, the precentral gyrus serves as the primary motor command center, orchestrating voluntary movement execution through its connections to the basal ganglia^16,33–35^. Collectively, these findings suggest that bradykinesia in PD may arise from reduced motor command efficiency in the precentral gyrus, disrupted sensorimotor feedback loops in the banks of the superior temporal sulcus and impaired multisensory integration in the inferior parietal lobule.

Dysfunction within the temporo-occipital visual network has been linked to impaired visual processing in PD, as demonstrated by neuroimaging studies^26,30,36^. The identification of the lateral occipital cortex, middle temporal gyrus, and lingual gyrus—key nodes of the temporo-occipital visual network—as regions associated with rigidity suggests that altered visual processing may be involved in the development of rigidity in PD. Deficits in visual processing within this network could disrupt sensorimotor integration, potentially leading to abnormal motor regulation and increased muscle tone, which may contribute to rigidity.

Tremor severity is linked to structural alterations in cortical and limbic regions. The strongest association (rank 1: right posterior cingulate curvature, 2.531) implicates disrupted sensorimotor integration and default mode network modulation contributed to tremor manifestation, consistent with previous report^37^. Inferior parietal thickness (rank 2: left hemisphere, 2.390) and pars opercularis thickness (rank 3: right hemisphere, 2.232) suggest aberrant motor planning and cognitive executive function were associated with tremor^28,32^. Involvement of the right banks of the superior temporal sulcus, bilateral lateral occipital curvature and parietal-occipital GMV may reflect visuospatial processing deficits influencing tremor amplitude^30,38^.

PIGD severity exhibits the most robust structural correlations with fusiform gyrus curvature, banks of the superior temporal sulcus thickness, and inferior temporal gyrus GMV. These findings suggest that PIGD pathophysiology involves disruptions in visuospatial integration deficits (mediated by the fusiform and inferior temporal gyri) and sensorimotor feedback dysfunction (attributed to banks of the superior temporal sulcus thickness impairment)^30,36^. The observed structural alterations align with PD’s progressive neocortical involvement, particularly in associative visual processing regions, which may underlie the complex interplay between motor and visuoperceptual impairments in PIGD phenotypes.

The PLSR models demonstrated distinct neuroanatomical substrates underlying non-motor symptoms in PD, with robust predictive accuracy across cognitive, and affective domains. Specifically, the right pars opercularis cortical thickness and left inferior parietal thickness align with prior evidence linking frontoparietal network integrity to cognitive executive function in PD^25,39^. Left inferior parietal thickness aligns with its role in sensorimotor integration and spatial awareness^32^. For anxiety (HAMA), the prominence of the right fusiform gyrus curvature and left rostral anterior cingulate thickness implicates limbic-temporal dysregulation. The fusiform gyrus, a hub for visual-emotional processing, may mediate anxiety through disrupted perception, while anterior cingulate involvement could reflect impaired emotional conflict resolution^30,40^.

Similarly, depression (HAMD) was strongly associated with left precuneus GMV (VIP = 2.416) and left rostral anterior cingulate thickness (VIP = 2.109), highlighting self-processing operations dysfunction and emotional regulation deficits in PD-related depression^40–43^. The involvement of hippocampal neuroplasticity/neurogenesis supports the “limbic-cortical” model of depression^44,45^. Non-motor symptom burden (MDS-UPDRS Part I) was driven by the right fusiform curvature and left entorhinal thickness, emphasizing the interplay between visuospatial processing (fusiform) and medial temporal integrity (entorhinal cortex). These findings resonate with Braak staging theory, where early α-synuclein pathology in olfactory/medial temporal regions propagates to cortical areas, exacerbating non-motor symptoms^5^.

Several limitations of the present study should be acknowledged. The cross-sectional design prevents causal inferences about structural brain changes and clinical progression in PD. Future longitudinal studies are therefore needed to clarify the temporal dynamics of cortical morphological changes and their predictive value for disease progression. Second, although the sample size was suitable for machine learning analyses, it may restrict the generalizability of the current findings. Moreover, feature selection, hyperparameter tuning, and model training were conducted within the same dataset, which may induce overfitting and limit model generalizability. This analytical framework may also partially explain the relatively low specificity of 54% observed in our classification model. Future studies should recruit larger multi-center cohorts and perform external validation to enhance the robustness and clinical translatability of the identified biomarkers. Third, the present study only incorporated structural MRI-derived cortical metrics. To further improve differential diagnostic performance, subsequent models could integrate structural metrics with multi-modal imaging data, including resting-state functional MRI, diffusion tensor imaging, and quantitative susceptibility mapping.

This study advances the field of PD diagnostics by integrating 7 T MRI-derived multidimensional morphological features with machine learning, achieving high diagnostic accuracy (0.80). PLSR analyses further revealed robust associations between specific SVM-derived features and motor/non-motor symptoms, providing novel insights into PD’s structural underpinnings. These findings highlight the potential of combining high-resolution imaging and machine learning to identify precise, pathophysiological informed biomarkers for PD.

## Methods

### Participants

This prospective cohort study enrolled 98 PD patients and 74 healthy controls (HC) at Chinese PLA General Hospital from March 2022 to July 2023. PD diagnosis was independently confirmed by two neurologists using the Movement Disorder Society (MDS) Clinical Diagnostic Criteria^46^. The inclusion criteria for patients with PD were as follows: (1) Absence of major systemic disorders (e.g., cardiopulmonary insufficiency, severe renal/hepatic dysfunction). (2) No history of cerebrovascular pathology or intracranial neoplasms. (3) No Parkinson-plus syndromes or other neurodegenerative conditions. (4) No severe cognitive impairment. (5) No active psychiatric comorbidities. (6) No history of functional neurosurgery (e.g., deep brain stimulation, ablation therapy). (7) No substance dependence. (8) No contraindications for MRI scanning (e.g., metallic implants). (9) No adjustments to their anti-Parkinsonian medication regimens (≥1 month prior to study enrollment and MRI scanning). The inclusion criteria for healthy controls were as follows: (1) No history of neurological or psychiatric disorders. (2) No family history of PD or other neurodegenerative disorders among first-degree relatives. (3) No personal or familial history of psychiatric disorders. (4) Absence of major systemic illnesses. (5) No history of neurological conditions (e.g., traumatic brain injury, cerebrovascular events, intracranial neoplasms, cognitive impairment). (6) No prior neurosurgical interventions. (7) No contraindications for MRI scanning. (8) No history of substance abuse. Only right-handed participants were included. The study protocol received ethical approval from the Institutional Review Board of Chinese PLA General Hospital (approval number omitted for anonymity) and was prospectively registered at ClinicalTrials.gov (NCT06449404). Written informed consent complying with the Declaration of Helsinki was obtained from all participants.

### Clinical evaluation

PD patients underwent comprehensive motor and non-motor evaluations using the complete MDS-Unified Parkinson’s Disease Rating Scale (MDS-UPDRS), including Part I (non-motor daily living experiences), Part II (motor aspects of daily living), Part III (motor examination), and Part IV (motor complications). Motor domain subscores (bradykinesia, rigidity, tremor, postural instability/gait difficulty [PIGD]) were calculated from specified MDS-UPDRS III items (3.4–3.8; 3.3; 3.15–3.18; and 3.10–3.12) following established protocols^47,48^. Both PD patients and HC completed the Mini-Mental State Examination (MMSE), Hamilton Anxiety Scale (HAMA), and 17-item Hamilton Depression Rating Scale (HAMD). Demographic parameters (age, gender) were recorded for all participants, while PD-specific clinical data encompassed disease duration, levodopa equivalent daily dose (LEDD), and Hoehn & Yahr (H-Y) stage.

### MRI acquisition

All participants underwent single-timepoint 7 T MRI scanning using a MAGNETOM Terra system (Siemens Healthineers) equipped with a 32-channel phased-array head coil. High-resolution T1-weighted anatomical images were acquired through a magnetization-prepared 2 rapid gradient echoes (MP2RAGE) sequence with the following technical specifications: repetition time (TR) 4300 ms, echo time (TE) 2.27 ms, flip angle 4°, field of view 240 × 240 mm², isotropic voxel resolution 0.75 mm³, 192 consecutive sagittal slices (0.75 mm thickness).

### Preprocessing and morphometric measurements extraction for structural MRI data

The MP2RAGE sequence data were processed using FreeSurfer 7.4.1 (http://surfer.nmr.mgh.harvard.edu/) with the automated “recon-all” pipeline to generate morphometric measurements in native space, referenced to the Desikan-Killiany cortical atlas (see Supplementary Table S1 for regional details of this atlas). The pipeline incorporated motion correction, skull stripping, nonlinear surface registration, and segmentation of subcortical structures and gyral-sulcal boundaries. Morphological metrics comprised: GMV representing the aggregate volume of cortical structures; cortical thickness defined as the Euclidean distance between pial and white matter surfaces; curvature quantifying local folding complexity through surface geometry; surface area measuring the total exposed cortical mantle; and folding index calculated as the ratio of the pial surface area to its convex hull, reflecting global gyrification patterns.

### Feature selection

Statistical analyses were conducted using Python 3.9.25 with the statsmodels library (v0.14.4). Mixed-effects linear regression models were implemented through the MixedLM module to assess group differences in morphometric metrics across brain regions. Five distinct models were constructed, each corresponding to a specific neuroanatomical measure: GMV, CT, curvature, surface area, and folding index. Fixed effects included diagnostic group (i.e., PD patients vs. healthy controls), age, and sex, with total intracranial volume (TIV) incorporated as an additional covariate exclusively for GMV analyses. Subject-specific random intercepts were modeled to account for multiple structural measures across different brain regions within each individual. In each mixed-effects linear regression model, brain regions demonstrating significant group differences (*P* < 0.05) were subsequently selected as features for further analysis.

### Machine learning

A machine learning pipeline was implemented using Python’s scikit-learn library (version 1.3.2), incorporating data scaling and SVM classification^49^. Initially, features were standardized by centering to the mean and scaling to unit variance for both PD patients and healthy controls. The SVM model was optimized using three hyperparameters: the regularization parameter *C*, which balances margin maximization and classification error; the kernel coefficient *gamma*, which defines the kernel’s influence range; and the kernel type, chosen from linear, polynomial, or radial basis function (RBF). The RBF kernel, in particular, offers significant advantages for handling complex datasets. It can project data into a higher-dimensional space via the kernel trick, enabling the separation of non-linearly separable data^50^. Hyperparameter tuning was performed via grid search with 5-fold stratified cross-validation to evaluate parameter combinations systematically. The dataset was split into training and test sets using an 80–20 stratified split to preserve the original class distribution of PD patients and healthy controls. Model performance was evaluated on the test set using accuracy (reflecting overall classification correctness), F1-score (quantifying cross-class discriminative performance), and AUC (Area Under the Curve, assessing the model’s discriminative ability between groups). Feature importance was assessed using permutation importance with 10 repetitions, quantifying the performance degradation when feature values were randomly shuffled, thereby identifying their contribution to the model’s predictive accuracy^22^.

### Clinical associations

To investigate relationships between discriminative features identified through SVM analysis and clinical manifestations (motor/non-motor symptoms), Partial Least Squares Regression (PLSR) was independently applied to each clinical outcome^51^. SVM-derived features with permutation importance >0.01 served as predictors, while motor symptoms (MDS-UPDRS part III scores, bradykinesia, rigidity, tremor, postural instability/gait difficulty) and non-motor symptoms (MMSE for cognition, HAMA/HAMD for mood, MDS-UPDRS part I score for non-motor burden) were treated as response variables. The optimal number of latent components for each PLSR model was determined through 5-fold cross-validation to balance model complexity and predictive performance. Missing data for MMSE (in 12 patients), HAMA (in 24 patients), and HAMD (in 25 patients) were imputed using the mean value before modeling. Model significance was evaluated via F-tests comparing explained variance against null models. Correlations between predicted and actual clinical scores were evaluated using Pearson correlation analysis. Bonferroni correction was applied to correct for multiple comparisons, and results were considered significant at a threshold of *P* < 0.05/9 = 0.006. For statistically significant PLSR models, Variable Importance in Projection (VIP) scores were computed to quantify feature contributions to symptom variance^51^. Features surpassing the empirically validated VIP threshold (>1.5) were interpreted as clinically meaningful predictors, enabling systematic evaluation of biomarker-symptom relationships across motor and non-motor domains. This approach offers a robust framework for elucidating how specific features correlate with clinical manifestations in PD, thereby enhancing our understanding of disease mechanisms and potential therapeutic strategies.

### Other statistical analyses

Demographic and clinical characteristics were analyzed to assess differences between PD patients and HC. Continuous variables were evaluated using the two-sample independent t-test for normally distributed data (e.g., age) and the Mann-Whitney U test for non-normally distributed data (e.g., MMSE, HAMA, HAMD). For categorical variables (e.g., gender), the chi-squared test was employed. Statistical significance was determined using a threshold of *P* < 0.05.

## Supplementary information

Supplementary Tables

## Data Availability

The conditions of our ethics approval do not permit the public archiving of raw data. The datasets generated and/or analyzed during the current study are not publicly available due to the constraints imposed by our ethics approval but are available from the corresponding author on reasonable request.
