# Supplementary material for: Support vector machine-driven Parkinson’s disease identification: a 7-Tesla multidimensional structural MRI approach
Source: NPJ Parkinsons Dis. 2026 Apr 29;12:167. doi: 10.1038/s41531-026-01370-3 (PMC13333910; doi:10.1038/s41531-026-01370-3)
Supplement: Supplementary file 1 — Supplementary Tables [file 41531_2026_1370_MOESM1_ESM.pdf]

Supplemental tables:

Table S1. The Abbreviations and their corresponding Full English Names for the regions outlined in the Desikan-Killiany cortical atlas.

Abbreviation	Full English Name	Abbreviation	Full English Name
bankssts	banks of the superior temporal sulcus	parahippocampal	parahippocampal gyrus
caudalanteriorcingulate	caudal anterior cingulate cortex	parsopercularis	pars opercularis (inferior frontal gyrus)
caudalmiddlefrontal	caudal middle frontal gyrus	parsorbitalis	pars orbitalis (inferior frontal gyrus)
cuneus	cuneus cortex	parstriangularis	pars triangularis (inferior frontal gyrus)
entorhinal	entorhinal cortex	pericalcarine	pericalcarine cortex
frontalpole	frontal pole	postcentral	postcentral gyrus
fusiform	fusiform gyrus	posteriorcingulate	posterior cingulate cortex
inferiorparietal	inferior parietal cortex	precentral	precentral gyrus
inferiortemporal	inferior temporal gyrus	precuneus	precuneus cortex
insula	insular cortex	rostralanteriorcingulate	rostral anterior cingulate cortex
isthmuscingulate	isthmus of the cingulate cortex	rostralmiddlefrontal	rostral middle frontal gyrus
lateraloccipital	lateral occipital cortex	superiorfrontal	superior frontal gyrus
lateralorbitofrontal	lateral orbitofrontal cortex	superiorparietal	superior parietal cortex
lingual	lingual gyrus	superiortemporal	superior temporal gyrus
medialorbitofrontal	medial orbitofrontal cortex	supramarginal	supramarginal gyrus
middletemporal	middle temporal gyrus	temporalpole	temporal pole
paracentral	paracentral lobule	transversetemporal	transverse temporal cortex

Table S2. The features identified by Mixed-effects linear regression models.

Metric	Region	P_value
**GMV**		
1	lh_inferiortemporal	0.040
2	lh_precentral	0.040
3	lh_precuneus	0.016
4	lh_superiorparietal	0.006
5	rh_caudalmiddlefrontal	0.002
6	rh_lingual	0.013
7	rh_medialorbitofrontal	0.000
8	rh_middletemporal	0.005
9	rh_paracentral	0.001
10	rh_precentral	0.000
11	rh_precuneus	0.013
12	rh_rostralmiddlefrontal	0.023
13	rh_superiortemporal	0.004
**surface area**		
1	lh_cuneus	0.014
2	lh_superiorparietal	0.008
3	rh_paracentral	0.000
4	rh_parahippocampal	0.019
**CT**		
1	lh_entorhinal	0.045
2	lh_inferiorparietal	0.045
3	lh_rostralanteriorcingulate	0.047
4	rh_bankssts	0.000
5	rh_insula	0.000
6	rh_parsopercularis	0.000
7	rh_parstriangularis	0.010
**meancurv**		
1	lh_lateraloccipital	0.006
2	rh_fusiform	0.046
3	rh_posteriorcingulate	0.000
**foldind**		
1	lh_cuneus	0.004
2	lh_lateraloccipital	0.040
3	lh_superiorfrontal	0.000
4	lh_supramarginal	0.034
5	rh_paracentral	0.011
6	rh_parahippocampal	0.011
7	rh_rostralmiddlefrontal	0.022

GMV, gray matter volume; CT, cortical thickness; foldind, folding index; rh, right hemisphere; lh, left hemisphere.

Table S3. Fold-wise performance of SVM models in the training set.

Performance	Fold 1	Fold 2	Fold 3	Fold 4	Fold 5	mean±std
Accuracy	0.71	0.61	0.59	0.67	0.74	0.66±0.12
F1 Score	0.70	0.5	0.57	0.65	0.73	0.63±0.13
AUC	0.69	0.73	0.65	0.78	0.80	0.73±0.11

AUC, Area Under the Curve; std, Standard Deviation.

Table S4. The feature contribution of PLSR analyses for motor manifestations

Ranking	Feature	VIP
**MDS-UPDRS Part III score**		
1	rh_bankssts-thickness	2.200
2	rh_precentral-GMV	1.634
3	lh_inferiorparietal-thickness	1.521
**bradykinesia**		
1	lh_lateraloccipital-foldind	2.209
2	rh_bankssts-thickness	2.012
3	lh_inferiorparietal-thickness	1.602
4	rh_precentral-GMV	1.526
**rigidity**		
1	lh_lateraloccipital-foldind	1.594
2	rh_middletemporal-GMV	1.592
3	rh_lingual-GMV	1.562
**Tremor**		
1	rh_posteriorcingulate-curvature	2.531
2	lh_inferiorparietal-thickness	2.390
3	rh_parsopercularis-thickness	2.232
4	rh_bankssts-thickness	2.028
5	rh_parstriangularis-thickness	1.933
6	lh_lateraloccipital-curvature	1.924
7	lh_rostralanteriorcingulate-thickness	1.849
8	rh_parahippocampal-area	1.812
9	lh_lateraloccipital-foldind	1.693
10	lh_inferiortemporal-GMV	1.652
11	rh_lingual-GMV	1.586
12	lh_superiorparietal-area	1.563
**PIGD**		
1	rh_fusiform-curvature	1.854
2	rh_bankssts-thickness	1.740
3	lh_inferiortemporal-GMV	1.707
4	lh_entorhinal-thickness	1.616

GMV, gray matter volume; foldind, folding index; rh, right hemisphere; lh, left hemisphere.

Table S5. The feature contribution of PLSR analyses for non-motor manifestations

Ranking	Feature	VIP
**MMSE**		
1	rh_parsopercularis-thickness	2.008
2	rh_medialorbitofrontal-GMV	1.926
3	lh_inferiorparietal-thickness	1.713
**HAMA**		
1	rh_fusiform-curvature	2.321
2	lh_inferiortemporal-GMV	1.905
3	lh_rostralanteriorcingulate-thickness	1.538
**HAMD**		
1	lh_precuneus-GMV	2.416
2	lh_rostralanteriorcingulate-thickness	2.109
3	rh_parahippocampal-foldind	1.857
4	lh_inferiorparietal-thickness	1.777
5	rh_paracentral-area	1.665
6	rh_middletemporal-GMV	1.608
7	rh_precuneus-GMV	1.607
8	rh_medialorbitofrontal-GMV	1.605
9	rh_lingual-GMV	1.603
10	rh_superiortemporal-GMV	1.573
11	rh_paracentral-GMV	1.559
12	rh_parahippocampal-area	1.551
13	lh_inferiortemporal-GMV	1.512
14	rh_caudalmiddlefrontal-GMV	1.500
**MDS- UPDRS Part I score**		
1	rh_fusiform-curvature	2.453
2	rh_medialorbitofrontal-GMV	1.689
3	lh_entorhinal-thickness	1.680

GMV, gray matter volume; foldind, folding index; rh, right hemisphere; lh, left hemisphere.
